# Thermal infrared imaging for conveyor roller fault detection in coal mines

**DOI:** 10.1371/journal.pone.0307591

**Published:** 2024-07-22

**Authors:** Yang Xiuyu, Tian Liyong, Cai Feng

**Affiliations:** 1 School of Mechanical Engineering, Liaoning Technical University, Fuxin City, China; 2 China Coal Energy Group Co., Ltd., Beijing City, China; Asia University, TAIWAN

## Abstract

As a key mechanism of belt conveyor, the health status and working state of its parts have a profound impact on whether the belt conveyor can run normally and safely. In the composition of the standard belt conveyor, the number of rollers is numerous and scattered. At the same time, under the complex environment of the work site, the fault detection of each roller is particularly difficult. In order to solve the above problems, a diagnosis method based on thermal infrared image features is proposed to detect the faults of each roller mechanism in the belt conveyor. Firstly, the position of the idler is identified based on the YOLOv4 identification method, and then the sticking resistance and bearing damage of the idler are detected based on the temperature difference discrimination method. In this paper, the target recognition method based on YOLOv4 is used to identify the position of the roller, and the recognition accuracy is 93.8%, which meets the requirements of the project. The infrared image obtained by the dual-spectrum camera is used to distinguish the fault of the idler in the coal mine. The temperature of the bearing and surface of the normal roller increases rapidly within 10 minutes of operation, and the temperature changes slightly after 10 minutes of operation. The bearing damaged idler has a greater friction effect at the bearing, so the temperature at the bearing rises faster, and there is a temperature difference of about 7°C between the bearing and the normal roller. The surface temperature of the idler in the blocking state is also fast for about 20 minutes, and there will be a temperature difference of about 8°between the surface of the idler and the normal roller. In this paper, it is determined that the temperature rise coefficients of the roller surface and bearing under normal conditions are 24% 28% and 18% 22% respectively. It is determined that the threshold value of the temperature rise coefficient in the blocking state and the damaged state is 30% and 25% respectively, that is, when the surface temperature rise coefficient of the roller is detected to be more than 30%, it is determined that the card resistance fault occurs, when the temperature rise coefficient at the roller bearing is detected > 25%, the bearing damage fault is judged.

## Introduction

As one of the three main ways of industrial transportation, belt conveyor plays an indispensable role in modern production. The roller is an important mechanism to support the conveyor belt and materials, and the failure of the conveyor caused by the damage of the roller is one of the most common fault types of belt conveyor [1]. If the idler fails, it will increase the energy consumption of the belt conveyor, and seriously it will lead to belt deviation, fracture, equipment damage and even fire, resulting in significant economic losses and even casualties. However, due to the large number and scattered distribution of rollers in the conveyor, and the complex dynamic load and environment during its work [2], it is relatively difficult to detect them by using the traditional equipment fault diagnosis method.

Thermal infrared image monitoring technology has been very mature in the field of industrial production [3]. The abnormal temperature of the fault parts is observed by the thermal infrared image sensor to achieve the purpose of fault diagnosis. This project proposes a coal mine underground belt conveyor roller abnormal monitoring system based on thermal infrared image characteristics, which realizes the inspection of the coal mine underground belt conveyor idler, and uses the depth learning technology to determine the image position of the idler. The infrared temperature measurement technology is used to monitor the roller temperature to realize the abnormal monitoring of the roller.

The traditional roller fault detection methods mainly include manual detection, sound signal detection, vibration signal detection and so on. In view of the low reliability and accuracy of belt conveyor roller fault detection, this project proposes a coal mine underground belt conveyor roller abnormal monitoring system based on thermal infrared image features. The target area of the idler is determined based on the YOLOv4 image algorithm, and the high quality idler image data is obtained by image filtering and enhancement, feature extraction and other processing methods, and the temperature change data of the key position of the idler is determined by combining the thermal infrared data. In the traditional equipment fault identification method based on infrared image, the equipment fault state is usually directly identified by the equipment temperature in the infrared image of the equipment. This method is easy to be misjudged due to the influence of ambient temperature, so the relative temperature difference method is used to distinguish the roller fault.

## Related work

Under the running condition of the conveyor, the obvious realization characteristics of the fault idlers are the abnormal sound emitted by the irregular vibration during the rotation of the idlers and the accumulation of a large amount of heat inside or on the surface of the rollers. Therefore, the general method to detect the fault of the idler is through the vibration sound and the surface temperature of the idler.

At present, many enterprise scientific research institutes and universities have carried out research on roller fault detection.

(1) based on the vibration sound of the idler during operation, the fault of the idler is detected.

Aiming at the problem of fault detection of idlers, at present, many scholars and scientific research institutions have used pickups to obtain abnormal sound in the process of idler transmission, based on the principle of acoustics, the computer processing technology is used to filter and Fourier transform the collected sound, and the running state of the idler is judged by analyzing the changing trend of sound pressure and sound spectrum.

The vibration signal detection method requires a contact sensor, which is not easy to collect data in a harsh environment, and the reliability of the collected signal is poor, and the vibration sensor is easy to be damaged [4,5].

In reference [6], by analyzing the vibration sound signal of the roller bearing, the time domain and frequency domain characteristics of the vibration sound signal of the roller under various fault problems are analyzed, and the comparative experimental verification is carried out with this feature as a template. and then realize the fault detection and identification of the idler.

Reference [7] uses the combination of LabVIEW software and noise sensor to detect the running state of the idler. Firstly, the noise sensor is used to collect the noise information during the fault operation of the idler, and then the fault of the idler bearing is judged by analyzing the time domain characteristics of the noise information and the frequency domain characteristics after FFT conversion.

In reference [8], the sound signal is decomposed by variational mode decomposition, and the intrinsic mode function component is selected by the compound index of envelope entropy and kurtosis, and then the MFCC features are extracted, which increases the accuracy of roller fault diagnosis.

There is important fault information in the high frequency part of the roller fault bearing sound signal [9]. The use of Mel filter bank will result in the lack of MFCC features to characterize the roller bearing fault, resulting in a decrease in the accuracy of diagnosis.

On this basis, a roller fault diagnosis method based on Mel frequency cepstrum coefficient (MFCC) and gradient lifting decision tree is proposed in reference [10]. It is verified that the MFCC features in the sound signal of rollers can be used for fault classification, but the experimental environment is built with only one group of rollers running, and the noise interference of belt conveyor is not considered, which is a defect in most scholars’ research.

At present, this method can only detect the sound caused by the internal fault of the roller bearing and the loosening of the roller frame, so as to realize the fault detection and judgment of the roller [11]. Reference [12] uses the MFCC feature of multi-frame fusion to increase the effect of roller fault detection.

There are also some problems: first, in the process of sound processing, it is the most difficult to extract the fault sound of the idler and filter the ambient sound and other irrelevant sounds in the audio, and the processing process is also tedious and complicated.

Second, in the walking process of the patrol robot, whether this method can accurately locate the position of the fault idler needs to be further studied.

(2) detect the fault of idler based on the surface temperature of idler during operation.

When the idler fails, the idler moves irregularly, which leads to the local temperature rise caused by friction between the idler and the conveyor belt or inside the idler. The surface temperature of the idler is obviously higher than that of the idler under normal operation due to the friction for a long time. In view of the problems of roller damage and roller cylinder wear in reference [13,14], an optical fiber temperature measuring instrument is installed on the belt conveyor to collect the temperature change curve of the damaged roller, and take this as a reference to compare and analyze the temperature change curve of other rollers. the fault of idler is identified by the principle of two-dimensional normal distribution. According to the analysis of reference [15], after the roller is blocked, the temperature at the shaft end of the roller will rise to 80cm 100°C in a short time. The temperature sensor ZigBee wireless sensor module is installed on the roller shaft end. Combined with the advantages of ZigBee wireless sensor network technology and LabVIEW host computer system development software, a temperature detection system software is developed and designed. The reference [16] also adopts a similar method, installing temperature sensors and pressure sensors on the roller surface, using low-power equipment and detection modules to increase the service life of the equipment, and applying CSMA/CA wireless communication technology to reduce frequency hopping, distortion and interference in the communication process. In reference [17], the optical fiber distributed system structure is adopted to develop the equipment and detection system for roller temperature detection of belt conveyor, which can effectively reduce the incidence of fire and other accidents of belt conveyor in coal mine. At present, many enterprises and scientific research institutes also use optical fiber temperature measurement technology, infrared laser temperature measurement technology, memory alloy temperature detection technology and other technologies, which are used in the field of temperature detection of conveyor rollers. Contribute to the cause of coal safety in China [18–21].

The conveyor is a long-distance conveying equipment, and there are many problems in the method of installing the temperature sensor for each roller, such as difficult wiring, sensor maintenance and so on. With the wide application of infrared thermal imaging temperature measurement technology in the industrial field, using infrared thermal imaging technology instead of complex wiring mode to detect the surface temperature of idlers is the most economical and desirable method. combined with the corresponding visual identification technology to detect fault idlers independently will be an important research direction.

## Abnormal monitoring system of belt conveyor idler

**Fig 1:**
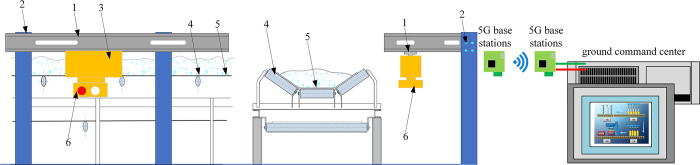
Layout of inspection robot for belt conveyor. 1-robot walking track; 2-walking track fixed frame; 3-conveyor inspection robot; 4-conveyor roller; 5-conveyor; 6-thermal imaging camera.

In this paper, the inspection robot is equipped with a thermal imaging camera. By assuming the track on one side of the belt conveyor and walking back and forth next to the belt conveyor, the thermal infrared image of the roller of the belt conveyor is collected in real time and the abnormal situation of the roller is analyzed. The layout of the belt conveyor inspection robot is shown in Fig 1.

Infrared thermal imaging technology is a method to obtain the temperature distribution of an object by detecting and converting the infrared thermal radiation emitted by the object. Its principle mainly includes two parts: the first part is the detection and conversion of infrared thermal radiation. In the infrared thermal imaging system, special optical components and photodetectors are used to convert the infrared thermal radiation emitted by the object into voltage signals. When infrared radiation is injected into the detector, the photodetector absorbs light energy, converts infrared radiation into electron energy, and then converts it into voltage signal. The size of the signal is related to the wavelength of infrared thermal radiation, and different wavelengths of infrared radiation are captured and converted by different photodetectors.

**Fig 2:**
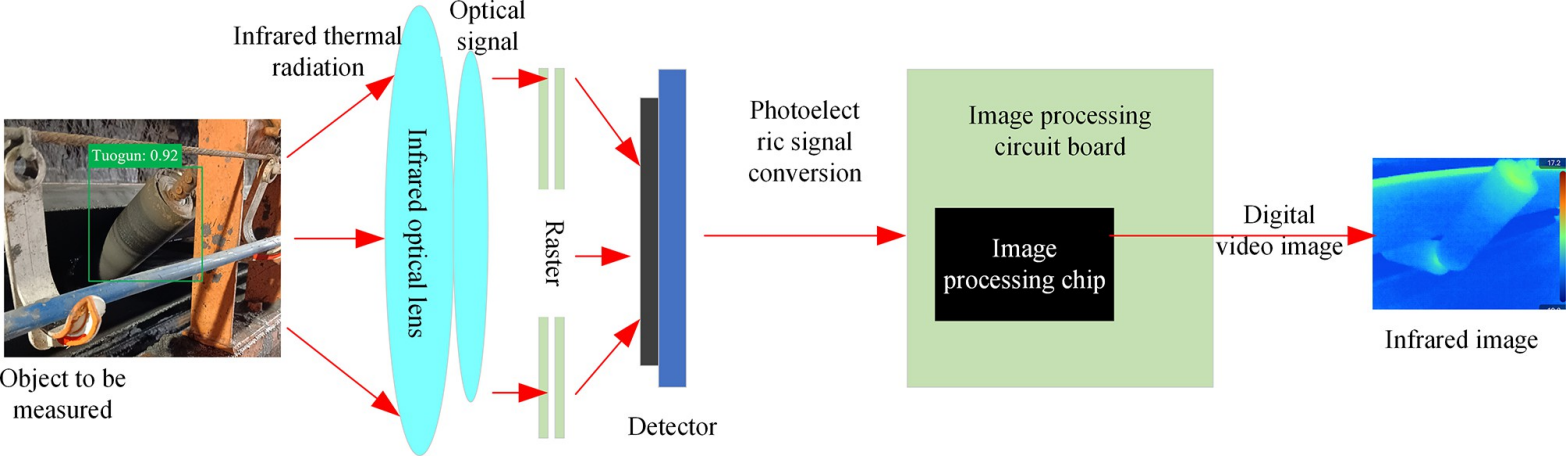
Schematic diagram of infrared thermal imaging.

The second part is the image processing and presentation of infrared thermal radiation. The infrared image system also includes an image circuit board and a processing chip. Through the image circuit board, it processes and amplifies the electronic signal of infrared radiation distribution obtained from the photodetector. The processing chip receives the amplified electronic signal and converts it into a digital video image signal. These image signals are then displayed by connecting to a monitor or display to form an infrared image. In this way, we can observe and analyze the temperature distribution of objects through visible light images, and realize the conversion from electricity to light. In general, infrared thermal imaging technology uses optical components and photodetectors to convert infrared thermal radiation into voltage signals, and converts them into digital video image signals through image circuit boards and processing chips. finally, it is presented on the monitor [22–24], and the infrared image which can reflect the temperature distribution of the object is obtained. The principle of infrared thermal imaging is shown in Fig 2.

**Fig 3:**
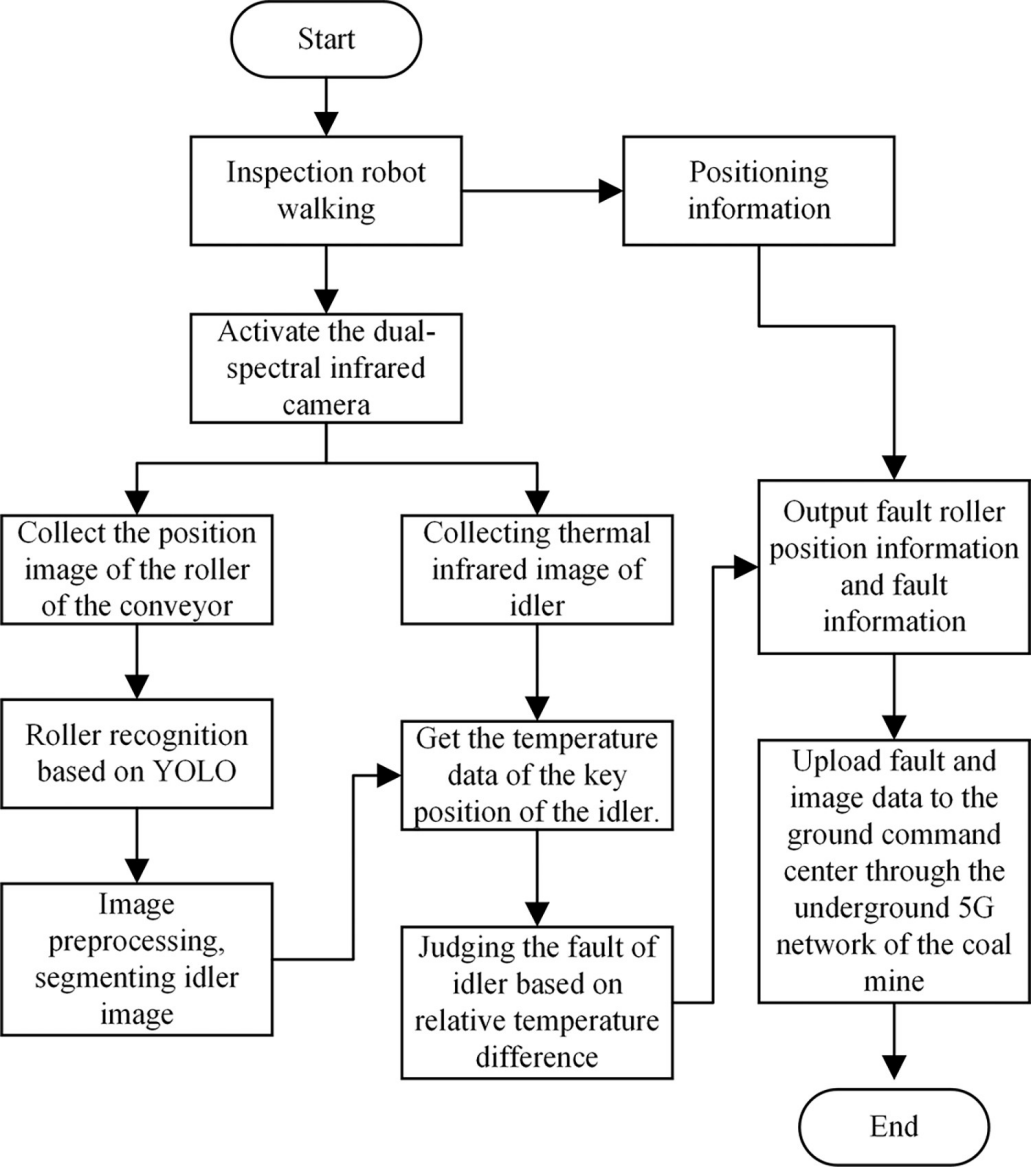
Abnormal monitoring flow of belt conveyor roller in coal mine based on thermal infrared image characteristics.

The traditional visible light single-spectrum camera can not detect the thermal infrared image and can not obtain the temperature data of the key position of the idler. The image collected by a single thermal infrared camera is not easy to detect the idler target. Therefore, this paper uses a dual-spectral camera to detect the abnormality of the idler. The abnormal monitoring work flow of belt conveyor roller in coal mine based on dual-spectral camera is shown in Fig 3.

First of all, the inspection robot walks back and forth on the conveyor and collects the image of the position of the conveyer roller by the dual-spectrum infrared camera. The image collected by the visible camera is used to identify the position of the idler, and the YOLOv4 algorithm is used to detect the idler target in this paper. The infrared image of the idler collected by the thermal infrared camera is used to obtain the temperature data of the key position of the idler. Combined with the temperature data of the visible light camera and the key position of the idler, the abnormal situation of the idler can be judged. The relative temperature difference method is used to judge the fault type of the idler. Finally, combined with the positioning information of the inspection robot, the fault roller position information and fault information are output.

**Fig 4:**
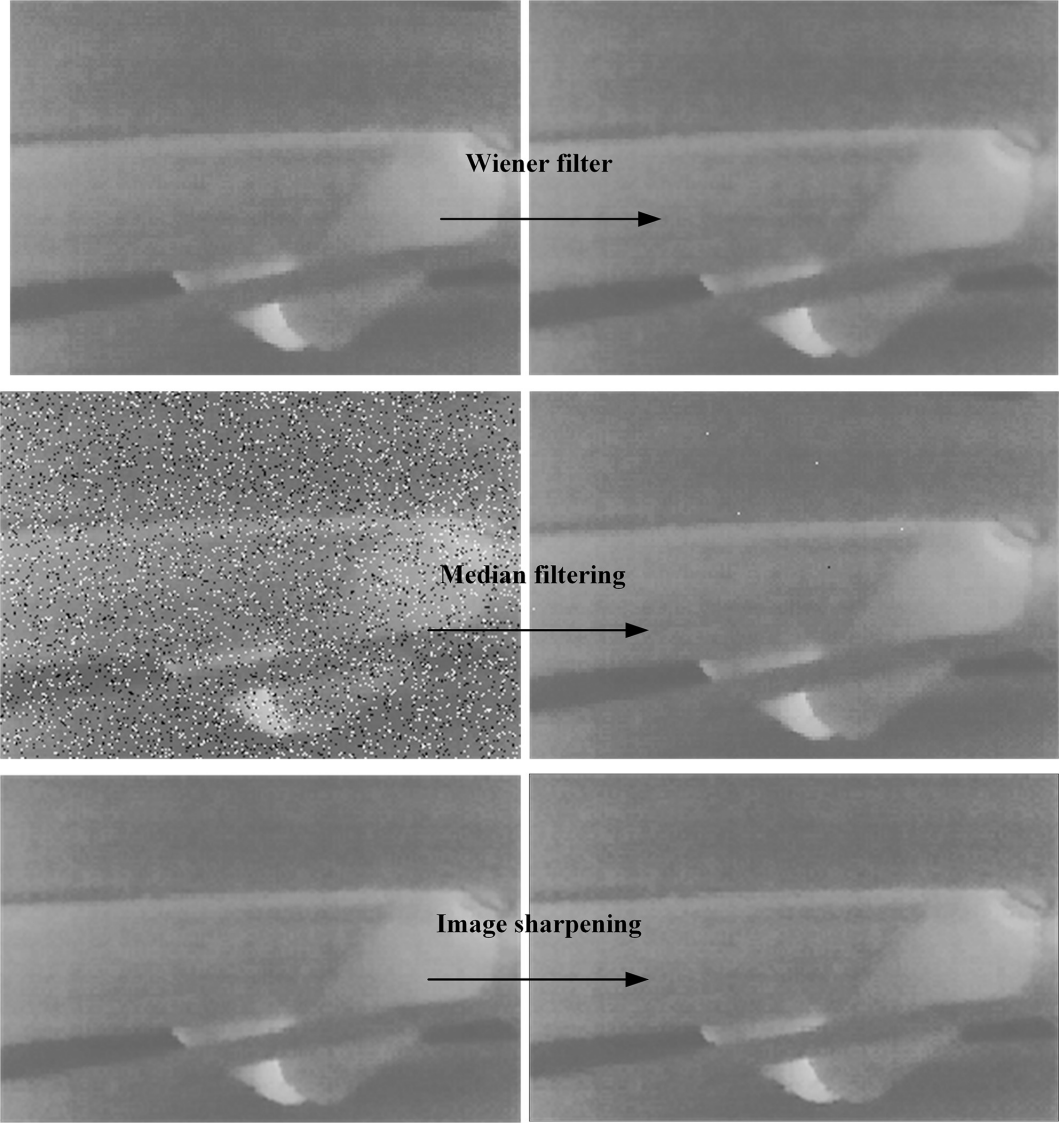
Result of image preprocessing.

Considering the influence of light, dust and idler movement on image acquisition in coal mine, Wiener filter is used to filter the motion blur and Gaussian white noise of thermal infrared image. adaptive median filtering is used to suppress the noise in the thermal infrared image, and adaptive histogram equalization and image sharpening algorithms are used to enhance the image. The effect of image preprocessing is shown in the Fig 4.

Wiener filtering is usually used to extract useful signals polluted by noise, filter according to the minimum mean square error criterion, take the correlation matrix of image and noise as the optimal criterion, and adjust the filtering output according to the local variance of the image.

By comparing the pixel values in a certain field, the median filter takes the median value as the central pixel value in this field.

According to the preset conditions, an adaptive median filter is selected to dynamically change the window size to reduce the impact of noise on the image and protect the image details.

Adaptive histogram equalization and Roberts image sharpening algorithm are used to process the thermal infrared image of the roller, enhance the non-correlation between the target roller region and the background region, improve the local contrast of the image and obtain more image details.

## Position recognition of idlers based on deep learning

The inspection robot walks back and forth on the conveyor and collects the image of the position of the conveyer roller by the dual-spectrum infrared camera. For the conventional image, the YOLO algorithm is used to recognize the idler image, then the image preprocessing method is used to segment the idler image and put forward the idler image features.

**Fig 5:**
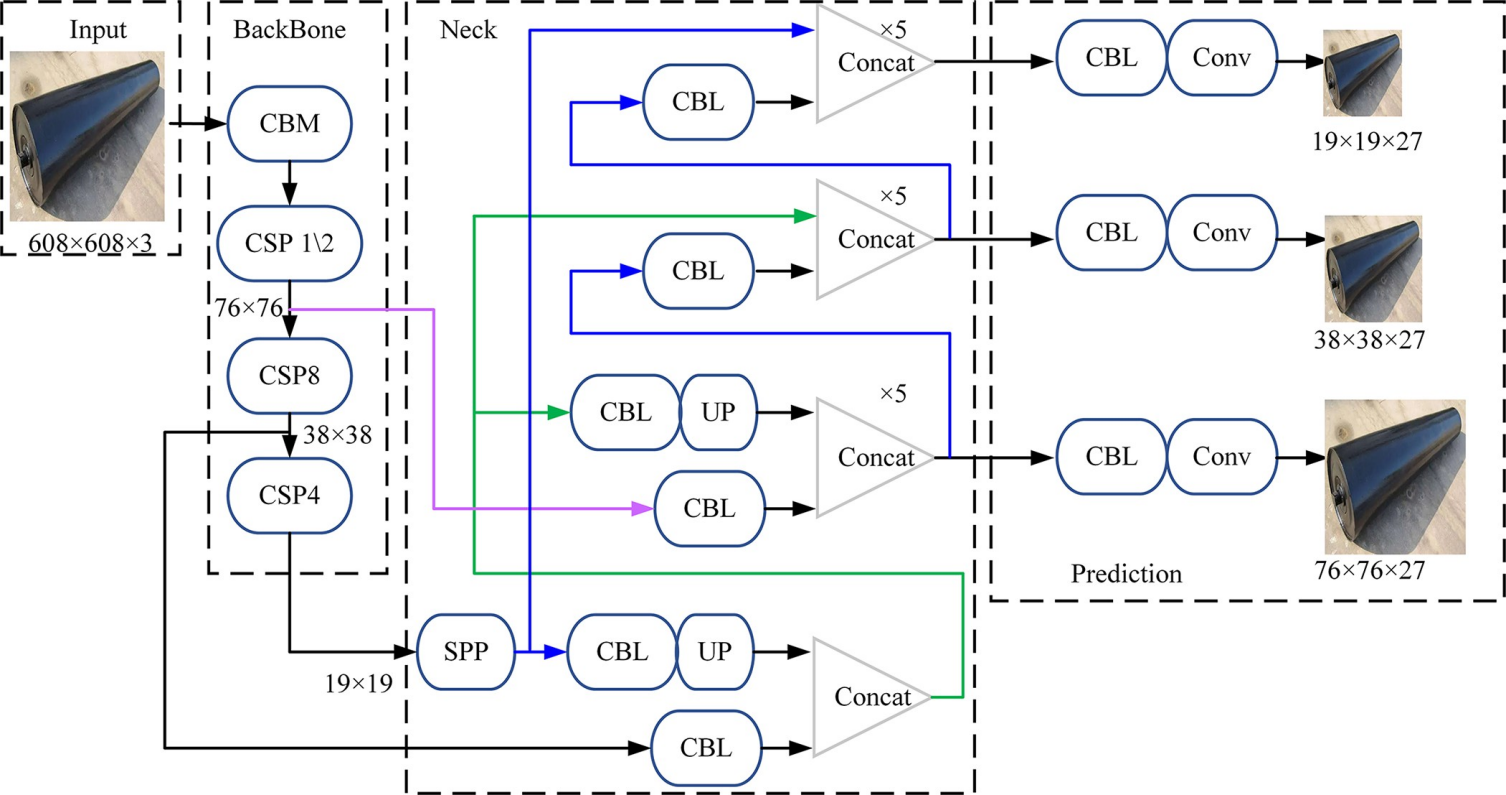
Structure of idler identification and inspection model.

YOLO is a target detection algorithm based on convolution neural network, which has been widely used in the field of image recognition and target detection in recent years. In this paper, YOLO V4 model is used to identify idlers. Compared with the previous generation YOLO V3 detection model, the network structure of this model is more complex, because it integrates more efficient training algorithms in the input layer, so it also has higher recognition efficiency [25]. The model consists of input layer, head backbone network CSPDarknet53, neck feature fusion network PANet, additional module SPP and head target recognition and frame regression network YOLO head. The overall architecture is shown in Fig 5.

Firstly, the head backbone network CSPDarknet53 is used to extract the input image features in the detection model studied in this paper, and the input network size is 608x608x3. Then the neck feature fusion network is used to generate three target feature images with different scales, and the feature image sizes are 76 × 76 × 27, 38 × 38 × 27 and 19 × 19 × 27. Different sizes detect different target objects. The feature image is divided into S × S grid [26]. Each grid needs to budget 27 bounding boxes and their probabilities belonging to different target objects. Finally, the confidence information of the boundary box including the foreign body target and the prediction of the foreign body target is output to the output layer.

**Fig 6:**
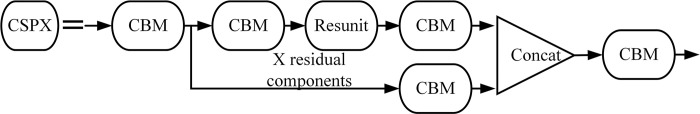
CSPNet structure.

The head backbone network CSPDarknet53 is used for target feature extraction, and it is also the core of the target detection model. It has 5 large residual blocks and corresponding 1, 2, 8, 8 and 4 small residual blocks. Through the introduction of cross-stage peer-to-peer network CSPNet in large residual blocks, it has the advantages of both lightweight and accuracy. The CSPNet structure is shown in Fig 6.

The target feature mapping is divided into two groups: the convolution operation and the fusion of the last convolution result. By using the set of cross-gradient hierarchical structure, the waste of resources caused by repeated learning gradient information is avoided, which not only lightens the model but also improves the accuracy of target detection.

The target object of this paper is the idler on the conveyor belt, which is very different from the original data set of YOLO. The original YOLO priori frame is obtained through open data clustering, including people, common objects and other detection targets, but it can not be directly applied to the detection targets in this paper [27]. Therefore, it is necessary to generate a priori box suitable for the roller detection data set. K-means is a commonly used clustering algorithm, which classifies the data with higher similarity into the same category and the data with lower similarity into different classes according to the similarity criterion. Euclidean distance, cosine similarity and vector representation are often used to measure the distance between data. K-means uses the sum of squares of errors as a measure of goal differences:

$$SE=\sum_{i=1}^{k}\sum_{x\in C_{i}}{\left(C_{i}-x\right)}^{2}$$ (1)

In the formula, *C*_*i*_ is the center of clustering, and *x* is the data point belonging to class C.

## Abnormal discrimination of idler based on infrared image

**Fig 7:**
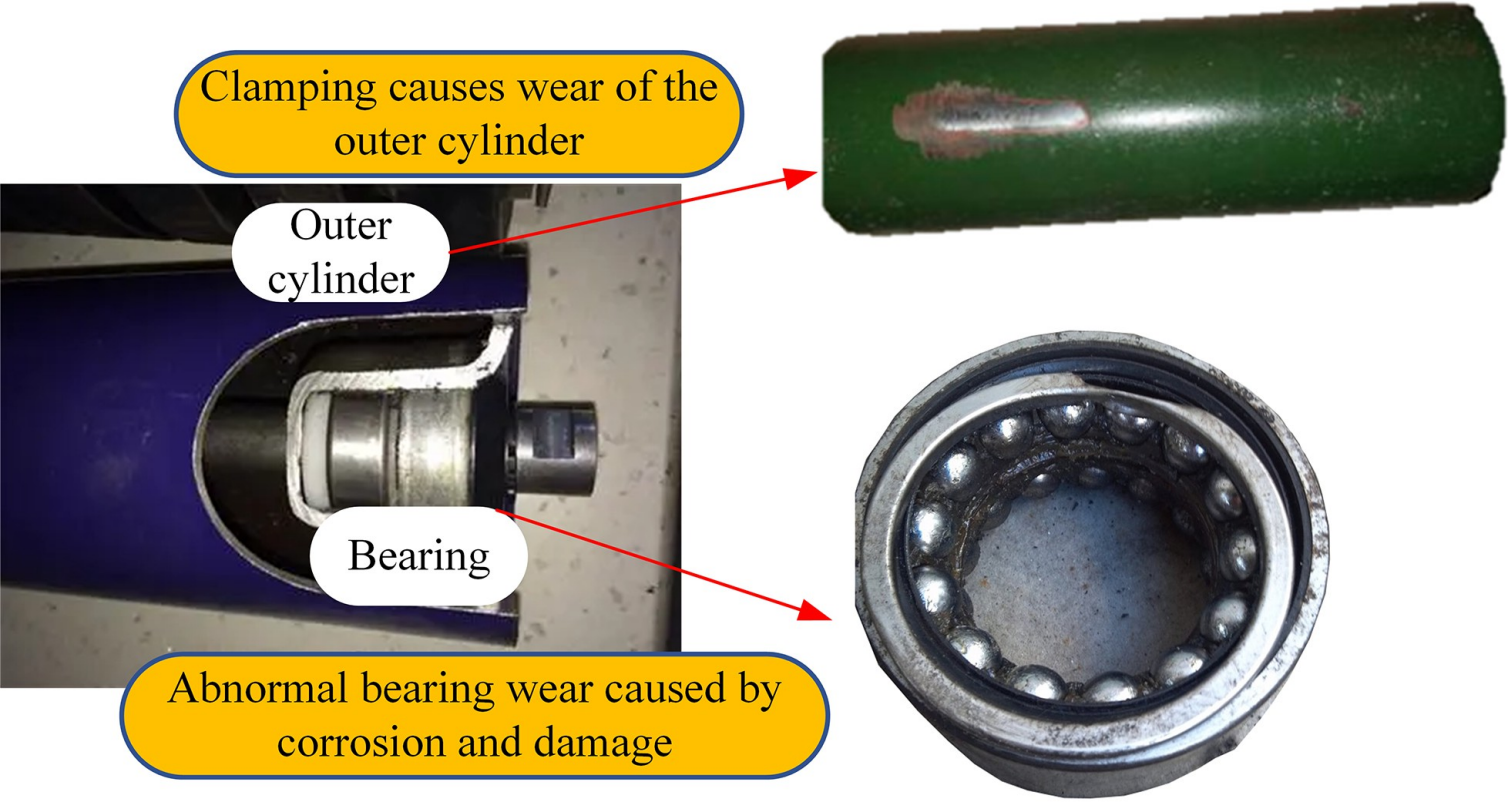
Failure types of conveyor rollers.

The common fault of belt conveyor idler is the wear of the outer cylinder caused by sticking, and the outer cylinder does not rotate, and the long-term friction with the conveyor belt will cause the temperature of the outer cylinder to rise, which can be distinguished by monitoring the temperature of the outer cylinder. Another fault is that the bearing is prone to rust and overload when it is used in the complex underground environment for a long time, which will cause abnormal wear of the roller bearing. The long-term wear will increase the temperature of the bearing, which can be judged by monitoring the temperature of the roller bearing. The failure types of conveyor rollers are shown in Fig 7.

In the traditional equipment fault identification method based on infrared image, the equipment fault state is usually directly identified by the equipment temperature in the infrared image of the equipment. This method is easy to be misjudged by the influence of ambient temperature, so this paper uses the relative temperature difference method to distinguish the roller fault [28]. The relative temperature difference method collects the temperature in the fault-prone area and the temperature in the fault-free area. The relative temperature difference is calculated according to the following formula, and then the roller fault is identified by the relative temperature difference, that is, temperature rise:

$$\alpha =\frac{\tau_{1}-\tau_{2}}{\tau_{1}}\times 100\%$$ (2)

In the formula: *τ*_1_ is the temperature of the fault zone,°C and *τ*_2_ is the temperature of the fault-free zone,°C.

## Experimental study

The abnormal monitoring method of belt conveyor roller in coal mine based on thermal infrared image characteristics is verified by experiments.

The test target is DTL80-15-2 × 37 mine belt conveyor. The length of the conveyer is 1500m. It has been transformed into a frequency conversion drive, and the belt speed can be adjusted within the range of 2.5~5.0m/s. The type of conveyor belt is PVG680s and the bandwidth is 800mm. The diameter of the drive drum is 500mm. The diameter of the idler is 89mm, the distance between the upper rollers is 1500mm, and the maximum groove angle is 30°. The infrared image is collected in real time by KBA12R mine intrinsically safe thermal imaging camera. The horizontal definition of KBA12R mine intrinsically safe thermal imaging camera can reach 400TVL under environmental illuminance (50°300) Lux. The range of temperature measurement is-20°C ~ 250°C, and the temperature measurement error is 2°C.

The workstation of Windows system is selected as the test platform, and the CPU is I9 12900K. The GPU is NVIDIA 3060 Ti, and the memory is DDR4 128g. The software platform is Visual Studio2019, OpenCV3.4.0, CUDA version 10.0. The algorithm is based on Darknet framework. The workflow of the algorithm is shown in Fig 3.

### Idler position identification

As the underground environment of the coal mine is complex and changeable, in order to enable the system to achieve better recognition and detection results under a variety of working conditions in the coal mine. The image samples of idlers under different lighting, dust and photo angles were collected, and a total of 2000 image samples were obtained by network search, which were used for model training. After iterative training, the idler recognition model was obtained. Then through some test samples to test the recognition accuracy and recall rate of the training model, the feasibility of the detection model has been determined.

In order to make the test results more accurate, use the marking tool labelImg to mark the idler image and establish the data set. The quality of training image and training parameters are very important to the quality of model training. Normalize the size of all collected pictures to 608 × 608. The training parameters are as follows: Batch64, Subdivision16, 4000 iterations, weight attenuation coefficient 5e-4, learning rate 1e-3, momentum 0.949, activation function Mish, weight preservation interval 1e+3. For a large number of samples, it is easy to cause long training time, such as overshoot, overfitting or underfitting and other unfavorable phenomena. Therefore, it is necessary to pay attention to the changing trend of model loss function.

After the completion of the training, the training model needs to be tested, and the performance of the model training is usually measured by the indicators such as accuracy P, recall R, detection accuracy AP and mean average accuracy mAP.

**Fig 8:**
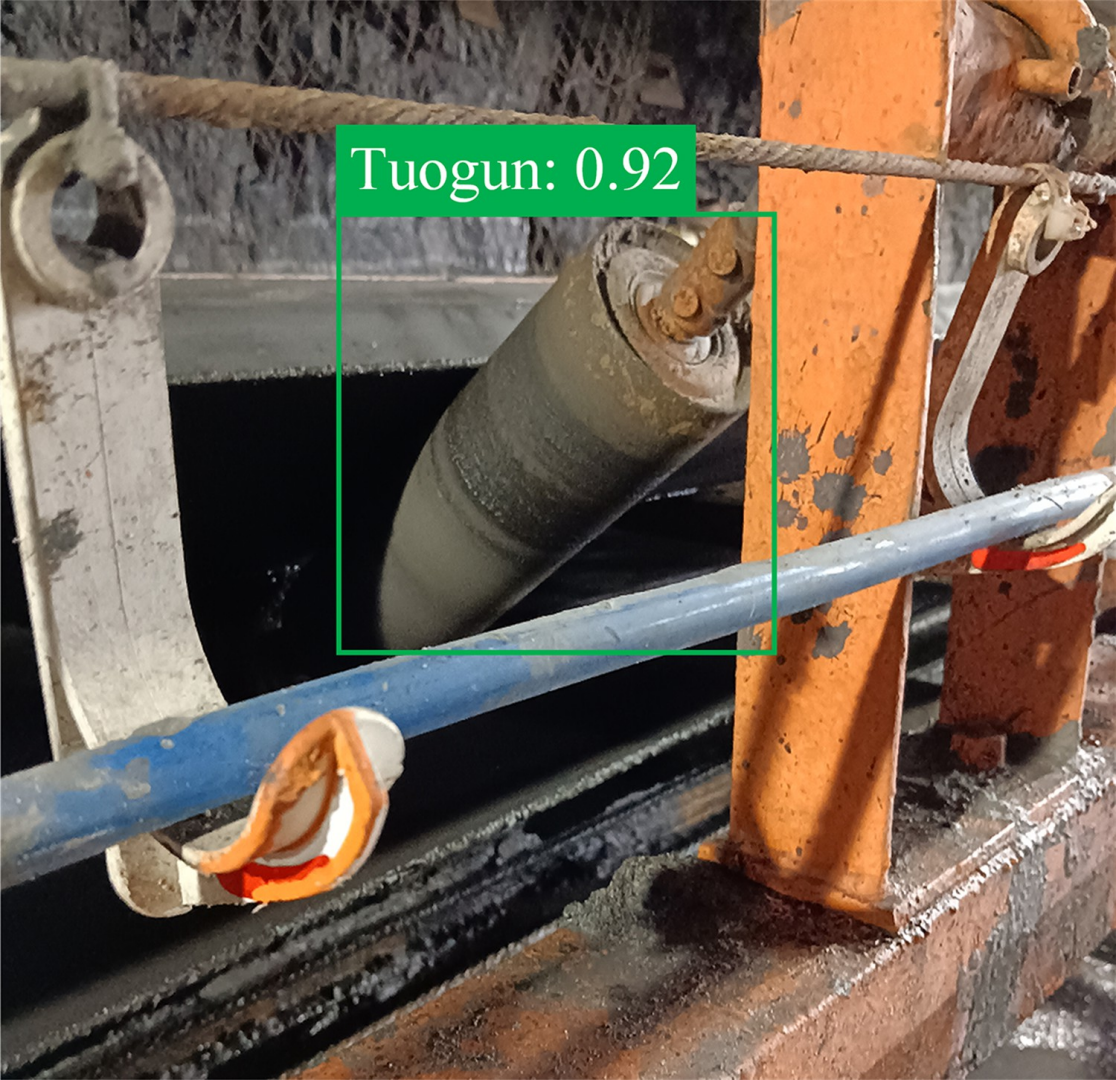
Target recognition of idler.

The test results show that the overall average detection accuracy of the detection model for the idler of the belt conveyor is 93.8%, and the error rate is 6.2%. It has a good identification accuracy and can basically meet the needs of the project, and the test results are shown in Fig 8.

It can be seen from the figure that the position of the idler can be accurately identified on the belt conveyor by using the idler detection method based on YOLO, and the frame regression effect is better.

### Fault detection of idler

**Fig 9:**
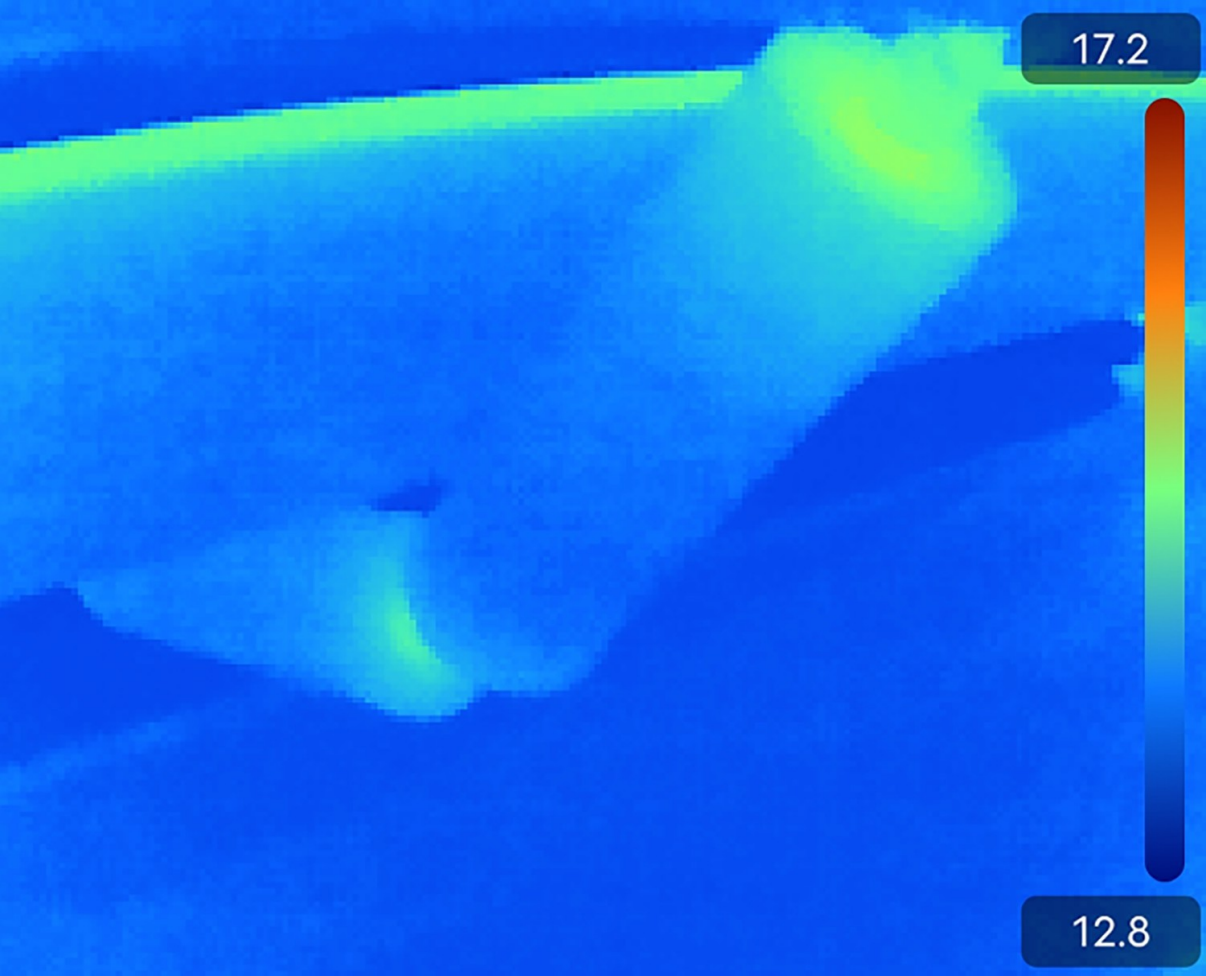
Infrared detection image of idler.

In the coal mine, the infrared image obtained by dual-spectrum camera is used to distinguish the fault of idler. First of all, the temperature at the normal roller bearing, the temperature at the damaged roller bearing, the normal roller surface temperature and the damaged roller surface temperature are detected in the coal mine, and the detection effect is shown in the Fig 9.

**Fig 10:**
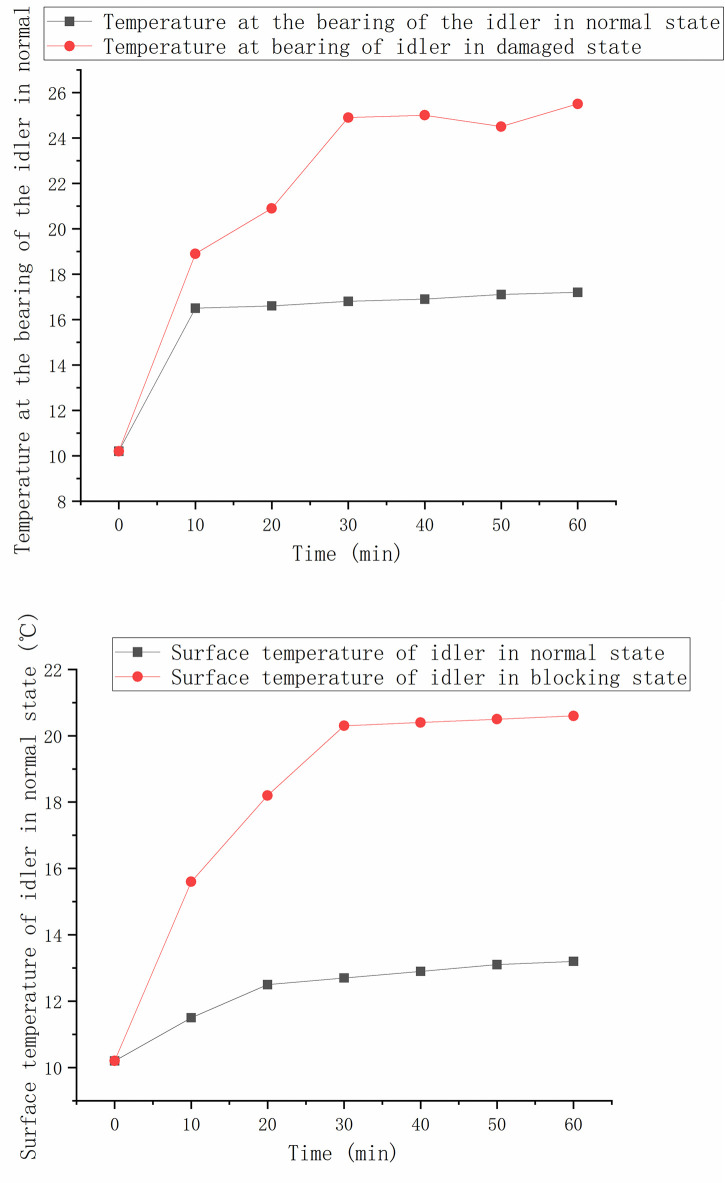
Temperature monitoring data of normal and faulty idlers. (a) Comparison of temperature at idler bearing. (b) Comparison of surface temperature of idler.

The temperature changes in the 60min of the conveyor are shown in the Fig 10.

It can be seen that the temperature of the normal idler rises rapidly within 10 minutes of operation, and the temperature changes slightly after 10 minutes. The bearing damaged idler has a greater friction effect at the bearing, so the temperature at the bearing rises faster, and there is a temperature difference of about 7°C between the bearing and the normal roller. The surface temperature of the idler in the blocking state is also fast for about 20 minutes, and there will be a temperature difference of about 8°between the surface of the idler and the normal roller.

**Fig 11:**
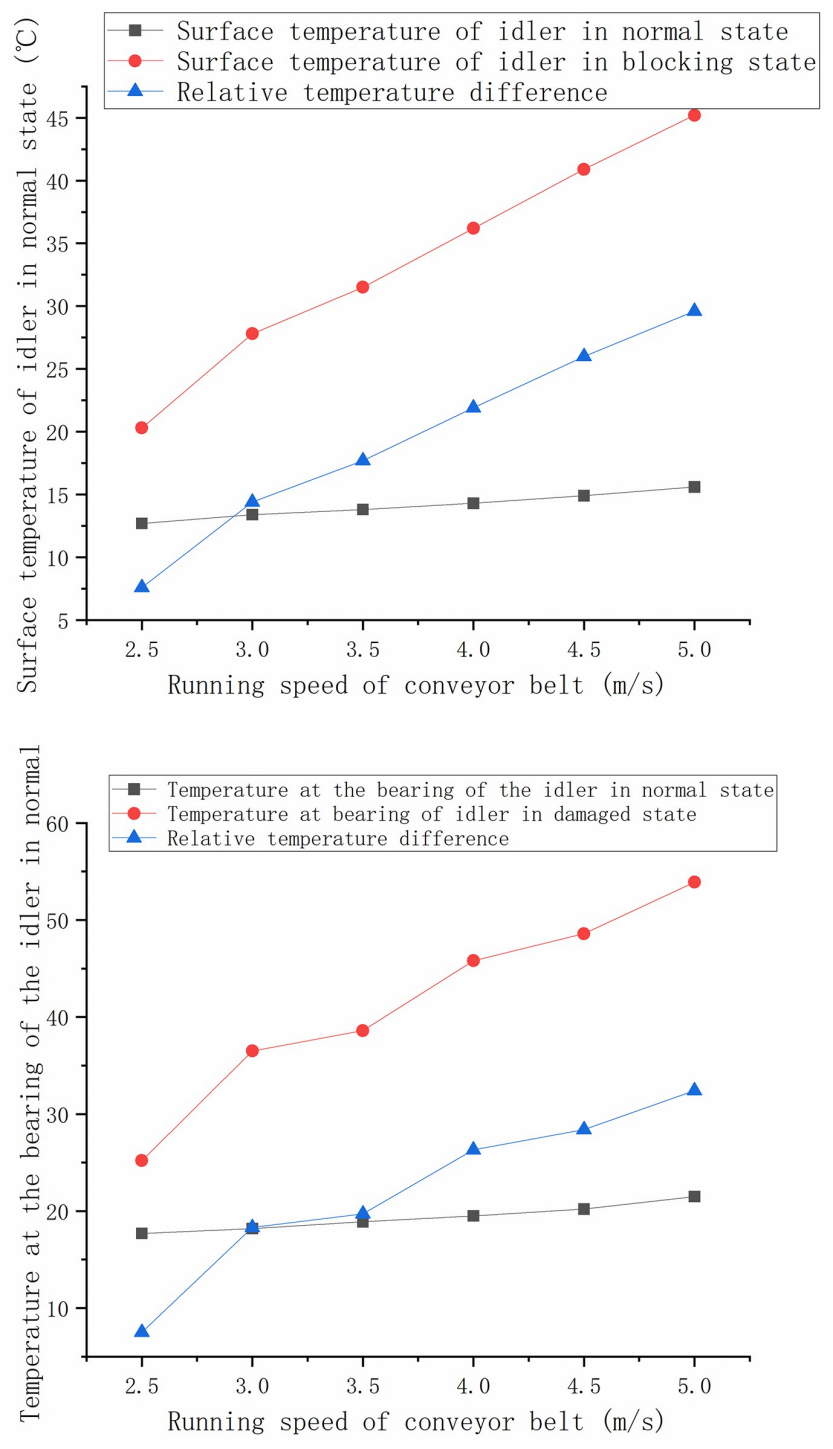
Temperature monitoring data of idlers at different belt speeds. (a) Comparison of temperature at idler bearing. (b) Comparison of surface temperature of idler.

Collect the normal roller bearing temperature, damaged roller bearing temperature, normal roller surface temperature and damaged roller surface temperature under different belt speed as shown in the Fig 11.

It can be seen that the bearing and surface temperature of the normal idler increases under different belt speed, but it does not change much. The temperature of the bearing and surface of the damaged idler changes greatly with the increase of belt speed, mainly because the greater the belt speed is, the more severe the friction between the conveyor belt and the roller surface is, and the more intense the friction is at the internal bearing of the idler, the faster the temperature rises.

The temperature rise coefficient of normal roller bearing, damaged roller bearing, normal roller surface temperature and damaged roller surface temperature under different belt speed is calculated according to Formula (2), as shown in Table 1.

**Table 1:** Temperature rise coefficient.

Running speed of conveyor belt/m/s	Bearing damage fault		Fault of roller clamping	
	Relative temperature difference /°C	Temperature rise coefficient /%	Relative temperature difference /°C	Temperature rise coefficient /%
2.5	7.6	37.4	7.5	29.8
3	14.4	51.8	18.3	50.1
3.5	17.7	56.2	19.7	51.0
4	21.9	60.5	26.3	57.4
4.5	26	63.6	28.4	58.4
5	29.6	65.5	32.4	60.1

According to the temperature rise data shown in Table 1, the infrared image of the idler is collected by a dual-spectral camera, and the sticking resistance and bearing damage faults of the idler can be accurately identified combined with the belt speed.

Usually considering the measurement error of the sensor, there will be a measurement error range of 3°to 5°when measuring the temperature of the normal idler. The temperature rise coefficient of the roller surface and bearing under normal conditions is determined by the maximum error of 5°C. The temperature rise coefficient of the roller surface is 24%-28%, and the temperature rise coefficient at the roller bearing is 18%-22%. Combined with the temperature rise coefficient everywhere under the fault condition in Table 1, it is determined that the threshold value of the temperature rise coefficient in the blocking state and the damaged state is 30% and 25% respectively, that is, when the surface temperature rise coefficient of the idler is detected to be more than 30%, the card resistance fault is judged to occur. When the temperature rise coefficient at the roller bearing is more than 25%, the bearing damage fault is determined.

## Conclusion

In this paper, a diagnosis method based on thermal infrared image features is proposed to detect the faults of each roller mechanism in the belt conveyor.

1. this paper uses the target recognition method based on YOLO to identify the position of the idler, and the recognition accuracy is 93.8%, which meets the requirements of the project.
2. the temperature of different parts of the roller under different faults is monitored, and the temperature of the normal roller remains basically unchanged after running for a period of time, but the temperature at the fault will rise rapidly.
3. the fault temperature difference of the idler under different belt speed is tested, and the bearing and surface temperature of the normal roller increases at different belt speed, but it does not change much. The temperature of the bearing and surface of the damaged idler changes greatly with the increase of belt speed, mainly because the greater the belt speed is, the more severe the friction between the conveyor belt and the roller surface is, and the more intense the friction is at the internal bearing of the idler, the faster the temperature rises. Combined with belt speed, the sticking resistance and bearing damage faults of idlers can be accurately identified.
4. A single detection method has its limitations. In future research, multiple signals such as sound, vibration and temperature will be considered to detect and predict the faults of idlers more accurately.

## Supporting information

S1 Data (XLSX)
